# Functional and Phylogenetic Characterization of Bacteria in Bovine Rumen Using Fractionation of Ruminal Fluid

**DOI:** 10.3389/fmicb.2022.813002

**Published:** 2022-03-25

**Authors:** Ruth Hernández, Maryam Chaib De Mares, Hugo Jimenez, Alejandro Reyes, Alejandro Caro-Quintero

**Affiliations:** ^1^Max Planck Tandem Group in Computational Biology, Department of Biological Sciences, Universidad de los Andes, Bogotá, Colombia; ^2^Animal Microbiology Laboratory, Agrodiversity Department, Corporación Colombiana de Investigación Agropecuaria – AGROSAVIA, Bogotá, Colombia; ^3^The Edison Family Center for Genome Science and Systems Biology, Washington University School of Medicine, Saint Louis, MO, United States; ^4^Departamento de Biología, Facultad de Ciencias, Universidad Nacional de Colombia, Bogotá, Colombia

**Keywords:** new species, ruminal fluid, fractionation, MAGs, small-sized bacteria

## Abstract

Cattle productivity depends on our ability to fully understand and manipulate the fermentation process of plant material that occurs in the bovine rumen, which ultimately leads to the improvement of animal health and increased productivity with a reduction in environmental impact. An essential step in this direction is the phylogenetic and functional characterization of the microbial species composing the ruminal microbiota. To address this challenge, we separated a ruminal fluid sample by size and density using a sucrose density gradient. We used the full sample and the smallest fraction (5%), allowing the enrichment of bacteria, to assemble metagenome-assembled genomes (MAGs). We obtained a total of 16 bacterial genomes, 15 of these enriched in the smallest fraction of the gradient. According to the recently proposed Genome Taxonomy Database (GTDB) taxonomy, these MAGs belong to Bacteroidota, Firmicutes_A, Firmicutes, Proteobacteria, and Spirochaetota phyla. Fifteen MAGs were novel at the species level and four at the genus level. The functional characterization of these MAGs suggests differences from what is currently known from the genomic potential of well-characterized members from this complex environment. Species of the phyla Bacteroidota and Spirochaetota show the potential for hydrolysis of complex polysaccharides in the plant cell wall and toward the production of B-complex vitamins and protein degradation in the rumen. Conversely, the MAGs belonging to Firmicutes and Alphaproteobacteria showed a reduction in several metabolic pathways; however, they have genes for lactate fermentation and the presence of hydrolases and esterases related to chitin degradation. Our results demonstrate that the separation of the rumen microbial community by size and density reduced the complexity of the ruminal fluid sample and enriched some poorly characterized ruminal bacteria allowing exploration of their genomic potential and their functional role in the rumen ecosystem.

## Introduction

Global meat and milk production will grow to 75 and 1,020 Mt, respectively, by 2030, responding to the high food demand by humans (Cooke et al., 2020; OECD, 2021). To face the continuous expansion of the livestock population, more efficient cattle production systems are required, particularly in those countries where the pastures are the primary resource for the ruminants (Puniya et al., 2015). Cattle depend on complex microbial consortia to ferment the plant material. The microorganisms that inhabit the gastrointestinal tract degrade plant components providing energy and microbial protein to the animal for meat and milk production (Morgavi et al., 2013; Ribeiro et al., 2016). Degradation of plant material entering the rumen is carried out by bacteria, fungi, and protozoa; in exchange, cattle obtain volatile fatty acids, proteins, and vitamins necessary to meet their nutritional requirements (Lee et al., 2000).

Studies carried out in the rumen for the last 60 years using classical cultivation methods and, more recently, cultivation-independent methods have revealed that our knowledge about the microbial composition and potential functional roles of many of the species in the rumen remains unclear. Henderson et al. (2015) postulated that similar bacteria were abundant in a range of ruminants across the globe, and among these, 70% of the described OTUs could not be assigned to a formally recognized genus, evidencing the large uncharacterized taxonomic diversity. In addition, fewer than 450 ruminal bacteria have been isolated and cultivated (Seshadri et al., 2018). Therefore, a more significant effort is required to understand their role, dynamics, and response to different nutritional conditions, which is fundamental to improving the productivity of ruminants.

Research regarding the ruminal microbiota has grown steadily together with the development of methods available to characterize microbial communities in general. From the early denaturing gradient gel electrophoresis (DGGE) (Watanabe et al., 2001; Schloss et al., 2011) and pulsed-field gel electrophoresis (Swain et al., 1996), technologies have rapidly evolved with the advent of next-generation sequencing methods. Currently, a standard method is the sequencing of a phylogenetic marker such as the *16S rRNA* gene from prokaryotic organisms (Janda and Abbott, 2007). However, it is also known that amplicon-based *16S rRNA* gene characterization is prone to many biases, starting with the selection of primers themselves (Schloss et al., 2011). Another recent approach is full shotgun sequencing of community metagenomes (Quince et al., 2017), which has the advantage of characterizing, simultaneously, taxonomic and potential functional diversity. This method has been used recently to assemble complete ruminal genomes (Puniya et al., 2015), thus expanding the diversity characterized beyond the culturable microorganisms. However, an important limitation is the cost due to the depth of sequencing required to obtain enough data to characterize members of this community, which are present at lower abundance. Recent research has demonstrated that a core of similar bacterial groups is present in ruminants worldwide (Henderson et al., 2015). This core comprises members of diverse taxa that have been classified at different taxonomic resolution, including genera, such as *Prevotella*, *Butyrivibrio*, and *Ruminococcus*, and similarly, members of families, such as Ruminococcaceae and Lachnospiraceae, and members of the orders Clostridiales and Bacteroidales (Henderson et al., 2015). Nevertheless, reported differences in ruminal microbiota across cattle breeds are associated with variables such as milk production, predisposition for acidosis disease, methane production, health state, and feed performance (Goldstone et al., 2010; Lee et al., 2018). These differences are often associated with changes in the abundance of the most prevalent bacteria in the rumen environment and the existence of bacteria that are specific to each breed (De Mulder et al., 2018). Our ability to detect these changes in abundance, and, more generally, microbial players that are present at lower frequencies, is restricted by depth of sequencing and biases of methods such as *16S rRNA* gene and shotgun libraries (Stewart et al., 2018), thus, yielding a limited view of bovine ruminal bacteria.

Given the methodological biases mentioned above, the functional diversity in the rumen has focused on the description of the role of the most abundant microorganisms, such as members of the genus *Prevotella* sp. (Nathani et al., 2015; Stewart et al., 2018), and on the characterization of carbohydrate active enzymes, such as cellulases and esterases, of those abundant microorganisms (Goldstone et al., 2010; Lee et al., 2018). Other researchers have studied the functional role of the bacterial community in the degradation of plant fiber (Mayorga et al., 2016) and the functional potential of rumen wall bacteria (Mann et al., 2018). However, limited studies have been carried out on the function of other less characterized microorganisms in the rumen (Zehavi et al., 2018), which shows the enormous lack of knowledge about their participation in the fermentation of plant fiber and the ruminal ecosystem.

To counteract some of those limitations, Hernández et al. (2021) developed a methodology to study the microbial diversity of rumen targeting a more varied spectrum of microbes using a sucrose density gradient (5, 10, 20, 30, 40, 50, 60, and 70% w/v). Briefly, the sample is centrifuged through the sucrose density gradients, and cells migrate to their corresponding density fraction. As a result, the smallest microorganisms are found in the less concentrated and dense fractions, while the largest microorganisms, such as protozoa and bacteria aggregations are found in the more concentrated and dense fractions of the gradient. This approach successfully showed, through the *16S rRNA* gene marker community characterization, that the less dense and concentrated sucrose fractions of the gradient (5, 10, and 20%) showed a significantly different bacterial composition to that in the more concentrated and dense fractions of the gradient, as well as to an unfractionated sample of ruminal fluid. It is important to highlight that all bacteria identified *via* the *16S rRNA* marker gene in the fractions of the gradient were also identified in the standard or unfractionated sample of ruminal fluid. However, their abundance in the unfractionated sample was low enough that it would not be possible to reconstruct their genomes from average depth shotgun sequencing. Conversely, poorly characterized bacteria were enriched in the fractions of the gradient, opening the possibility to characterize these genomes functionally and phylogenetically.

Building upon our previous work, this research aims to reconstruct uncharacterized bacterial genomes from the tropical creole Bon and Holstein breeds that are part of the complex ruminal ecosystem, particularly a group of small-sized microorganisms previously enriched in the 5% sucrose density gradient described in Hernández et al. (2021). We focused on unveiling the potential functions of these genomes and inferring the phylogenetic relationships to previously identified bacteria, prioritizing those that represent a higher taxonomic novelty (large phylogenomic distance to cultivated relatives). For this purpose, we performed shotgun sequencing of the 5% fraction of a sample of ruminal fluid from a cow of Bon and a bull of the Holstein breed and compared it with an unfractionated or total sample of ruminal fluid from these two animals. We hypothesized that the 5% fraction would allow us to reconstruct metagenome-assembled genomes (MAGs) from uncharacterized microorganisms with higher quality and completion than that obtained from the total rumen sample.

## Materials and Methods

### Sample Collection

The experimental design and sampling were described in detail in Hernández et al. (2021). In summary, samples of ruminal fluid from a cow of the Colombian cattle breed Bon (Bon-B) and a Bull of the Holstein breed were collected for analysis following a 16-h fasting and right after feeding (post-feeding). The animals were fed with grass *ad libitum* right before and after the 16-h fasting. The ruminal fluid samples were collected by cannula for the Holstein animal and using a nasogastric probe for the Bon one (Minor et al., 1977). The ruminal microorganisms in these samples were separated by size and density using a sucrose density gradient, which consisted of a step gradient made of different sucrose concentrations, namely: 5, 10, 20, 30, 40, 50, 60, and 70%, where the most concentrated solutions were at the bottom of a 50 ml polypropylene centrifuge tubes, and the less concentrated solutions were at the top of the tube. The ruminal fluid was added to the tube and centrifuged. During centrifugation (5,000 × *g*), the ruminal microorganisms migrated to the fraction that had the same density (Hernández et al., 2021). To compare the fraction with the total ruminal microbial community, an extra set of samples at fasting and post-feeding were collected; these samples were not separated into fractions.

### DNA Extraction

As observed in our previous work (Hernández et al., 2021), the 5% fractions of the Bon-B and the Holstein bull both showed statistically significant differences in bacterial composition in comparison with the total or standard sample of ruminal fluid and, thus, were chosen for further analysis *via* shotgun sequencing together with the total samples for comparison. The DNA of each of the fractions in either sucrose density gradients or total samples was obtained using the protocol described in Hernández et al. (2021). Briefly, 5 ml of the 5% fraction was centrifuged at 19,064 × *g* at 4°C for 1 h. Next, the pellet was washed twice with phosphate buffer solution (8 g NaCl, 0.2 g KCl, 1.44 g Na_2_HPO_4_, 0.245 g KH_2_PO_4_ dissolved in 800 ml, adjusting pH from 6.8 to 7 and filling to 1 L). The pellets of three replicates from the same sample were combined in 2-ml tubes for DNA extraction. Three aliquots of 500 μl of the total sample of ruminal fluid were also combined in a 2-ml tube, and 500 μl was used for DNA extraction. Then samples were heated at 65°C in a water bath for 5 min and frozen with liquid nitrogen. This procedure was repeated three times. Subsequently, DNA extraction was done using the kit ZR Fungal/Bacterial DNA MiniPrep™ (Zymo Research) according to the instructions of the manufacturer. The presence of DNA after extraction was verified by electrophoresis in a 2% agarose gel (w/v), and DNA concentration as well as the presence of contaminants and quality were measured using NanoDrop (Thermo Fisher Scientific).

DNA from the 5% fractions (post-feeding and after fasting) and one total sample for each animal, postfeeding (Holstein) and after fasting (Bon-B), were sent to the Genome Sequencing Center at the Edison Family Center for Genome Sciences and Systems Biology Washington University in Saint Louis, MO, United States, where shotgun libraries were built using the standard Illumina Nextera protocol and sequenced with NextSeq generating pair-end reads of 2 × 150.

### Bioinformatic Analysis

The quality of the sequences was checked using the program FastQC v 0.11.7 (Andrews, 2010). Nextera adapters and low-quality positions [with a Phred value < 20 and a length smaller than 50 nucleotides were removed using Trimmomatic v 0.38 (Bolger et al., 2014)]. Subsequently, BMTagger (Rotmistrovsky and Agarwala, 2011) was used to remove sequences from human and bovine origin. Clean sequences were assembled using Megahit v1.0 (Li et al., 2016) with default parameter values. Sequences for all samples belonging to the same animal (either Bon-B or Holstein) were co-assembled. In addition, separate assemblies for each of the six samples were also performed using the same tool. Clean reads for each sample were mapped back to the assembled contigs using Bowtie2 (Langmead and Salzberg, 2012).

### Metagenome-Assembled Genomes Generation

Three different binning programs were used to generate MAGs. These programs were used with default parameter values. For co-assemblies, bins were built using CONCOCT (Alneberg et al., 2013), while MaxBin2 (Wu et al., 2016) and MetaBAT2 (Kang et al., 2019) were used for individual assemblies. The bins generated by CONCOCT were refined using Anvio’s pipeline v6 (Eren et al., 2021) described in the Anvio’s User Tutorial for Metagenomic Workflow,^1^ while the bins generated by MaxBin2 and MetaBAT2 were refined using DASTool (Sieber et al., 2018); however, in general, no improvement in the original bins was observed with DASTool, and several of the original bins already had sufficient quality (combination of completeness and contamination) to be kept. DASTool results were only kept if they showed a significant improvement.

The completeness and contamination (redundancy) of each of the refined bins were assessed with the CheckM tool (Parks et al., 2015). Bins with completeness greater than 70% and contamination below 10% were included in a preliminary analysis. Then based on completeness and contamination values, the quality of each bin was calculated using the equation generated by Parks et al. (2015), which is quality = [completeness - (5 * contamination)]. Those bins with a quality value greater than 50 were selected as high-quality MAGs. Reads mapped to each sample for one of the high-quality MAGs were calculated using Bowtie2. With these data, the reads per kb of the genome per million of sequences (RPKM) were calculated to normalize the abundance of a MAG in each of the samples.

### Taxonomic Identification of the Metagenome-Assembled Genomes

Intrinsic parameters of the MAGs, such as the total length, the number of predicted proteins, average length, predicted proteins, coding density, number of contigs, N50, length of the longest sequence, GC content, and taxonomic assignment were obtained for each of the bins using MiGA (Rodriguez-R et al., 2018). In addition, MAGs were clustered using the MASH average nucleotide identity to check if they represented unique operational taxonomic units or a species using DREP (Olm et al., 2017). For this approach, a representative bin for each cluster was picked based on the best Linye quality, best combination of intrinsic parameter metrics, highest completeness value, and lowest contamination.

To identify the closest reported genome to our MAGs in public databases, we used the Genome Taxonomy Database (GTDB),^2^ and a compendium of MAGs of the rumen (RUGs) compiled by Stewart et al. (2019) and the complete rumen genomes reported in the Hungate project 1000 database (Seshadri et al., 2018). The program CompareM,^3^ which is based on average amino acid identity (AAI), was used to find the closest reported RUGs or genomes to our MAGs.

### Phylogenetic Placement of Each Metagenome-Assembled Genomes

Following the methodology outlined by Hug et al. (2016) to reconstruct one of the most widely accepted versions of the Tree of Life, genomes were mined for bacterial-type (P-type) ribosomal proteins L2, L3, L4, L5, L6, L14, L15, L16, L18, L22, L24, S3, S8, S10, S17, and S19 (all single-copy genes). Sequences for each of our MAGs and the closest MAGs identified based on AAI from the RUGs and Hungate project databases (as described in the section above) were added to the existing alignment of the tree of life using MAFFT (Katoh and Frith, 2012), with the following command: mafft –thread 12 –reorder –keeplength –maxambiguous 0.05 –addfragments. The final alignment comprised 3,131 genomes and 2,596 amino acid positions. A maximum likelihood tree was constructed using RAxML v. 8.1.24 (Stamatakis, 2006), under the LG plus gamma model of evolution (PROTGAMMALG) with 100 bootstraps. The best resulting tree together with its associated bootstrap support values was visualized in the Interactive Tree of Life (iTOL) interface online (Letunic and Bork, 2011) for annotation. To determine taxonomic novelty, the taxonomic assignment was performed by GTDB-Tk v1.1.0 (Chaumeil et al., 2020). GTDB-Tk classifies bacterial and archaeal genomes and identifies novel taxa by determining the phylogenetic placement and relative evolutionary divergence (RED) values of query genomes in the GTDB reference tree (Parks et al., 2018, 2019), using the GTDB-Tk data release number 89 (June 21, 2019).

### Functional Annotation

The rapid annotation subsystem technology (RAST) (Aziz et al., 2008) was used to perform a general functional annotation of each of the selected MAGs. The functional categories such as carbohydrates, vitamins and cofactors, and protein degradation were annotated in detail. In addition, the complete genomes of *Prevotella ruminicola* (GCF_000025925.1), *Prevotella bryantii* (GCF_000179055.1), *Ruminococcus albus* (GCF_000178155.2), and *Butyrivibrio fibrisolvens* (GCF_000209815.1) were included in this functional characterization, as complete references, together with the closest genomes identified *via* AAI from the RUGs database. Heatmaps were used to visualize the abundance and clusterization of the different functional categories in the MAGs, the selected list of complete genomes, and the selected RUGs. The heatmaps were generated using R studio using the library ggplot2 v.3.3.5 (ggplot2.tidyverse.org). We counted the number of genes that were detected by RAST in each functional category for each genome or MAG. Heatmaps were produced for each functional category based on raw counts. On the other hand, the dbCAN2 meta server (Zhang et al., 2018) was used to identify the set of carbohydrate-active enzymes (CAZy) encoded in the MAGs, and the hits obtained with the dbCAN CAZyme were analyzed through the domain HMM database (Finn et al., 2011). The glycosyl hydrolases (GH), transferases (TR), esterases (CE), carbohydrate-binding modules (CBMs), polysaccharide lyases, and enzymes with auxiliary activity were identified in the assembled MAGs. Raw CAZy counts were recorded for each family, and these were visualized in conjunction with the phylogenetic information of each bin.

## Results

### The Sucrose-Based Fractionation Resulted in the Recovery of More Metagenome-Assembled Genomes Than the Total Sample

The fractionation of the ruminal fluid samples previously described in Hernández et al. (2021), allowed us to enrich less studied bacteria in the rumen of the Colombian Creole Bon and Holstein breeds, adapted to tropical environments. Here, we reconstructed the genomes from a fraction of a sucrose density gradient through shotgun sequencing. We selected the 5% sucrose fractions at fasting and post-feeding, and total or unfractionated ruminal fluid sample for one animal of each breed because those fractions showed very different bacterial compositions using *16S rRNA* gene profiling. Shotgun sequencing of the fractionated and total fraction produced around 11–13 million sequences per sample, respectively. After the removal of low-quality human and bovine sequences, the number of sequences decreased 5%, on average (Supplementary Table 1).

The reads that passed the quality control and filtering were co-assembled independently for each animal. The Bon-B co-assembly resulted in 75,695 contigs longer than 1,000 bp. The percentages of reads used in this assembly were 32.9, 35.1, and 7.1% for samples Bon-B-5%-fasting, Bon-B-5%-post-feeding, and Bon-B-Total, respectively. The Holstein co-assembly resulted in 97,141 contigs longer than 1,000 bp. The percentages of reads used in this assembly were 59.3, 63, and 32.4% for samples Holstein-5%-fasting, Holstein-5%-post-feeding, and Holstein-Total, respectively. In addition, each sample was assembled individually. The percentage of reads of each sample used in these assemblies ranged between 5.9 and 58.1%. Co-assembly and individual sample assembly statistics are shown in Supplementary Table 2.

We used different binning programs and refining tools to generate the MAGs. We generated 452 bins that were used as input for the different software. The binning program CONCOCT was used with the refining tool Anvi’o, resulting in two bins for the Bon-B and five bins for the Holstein animal. MaxBin2 generated one bin for the Bon animal and 17 bins for the Holstein animal, and MetaBAT2 tool reconstructed three bins for the Bon and 13 bins for Holstein. Then, the bins generated by MaxBin2 and MetaBat2 were refined using DASTool, which resulted in four improved bins for Bon and 14 bins for Holstein. Only 49 of those bins fulfilled the requirement of completeness greater or equal than 70%, a contamination value below 10% according to CheckM, and a quality value greater than 50 (Parks et al., 2015; Supplementary Table 3). This set of 49 high-quality bins was deemed as MAGs.

As expected, most MAGs assembled were more abundant in the 5% fractions than in the total sample (Supplementary Table 3), with RPKM values varying between 99.7 and 0.02 for the 5% fraction and between 12.6 and 0.02 for the total sample, with only one MAG being enriched in the total sample. Interestingly, the MAGs were similarly abundant in both 5% fractions, regardless of whether they were taken after fasting or post-feeding. In terms of the animal, we observed that the majority (41 MAGs) were derived from the Holstein animal, while only eight were obtained from the Bon-B animal. Contigs statistics showed that genome size of the MAGs enriched in the 5% fractions was between 769,455 and 3,517,274 bp, while the one single MAG enriched in the total sample had a genome size of 5,093,283 bp, further indicating the nature of smaller genomes in the 5% fraction (Supplementary Table 3).

### Metagenome-Assembled Genomes Recovered From Small (5%) Fractions Belong to the Unexplored Ruminal Bacteria

MIGA and Anvi’o were used for the taxonomic classification of the bins (Supplementary Table 3) and to identify closely related genomes or MAGs present in the GTDB. The MiGA tool showed that the most similar genomes had AAI values between 30 and 40%. In addition, MiGA was only able to classify our MAGs to order or family level with statistical significance; in fact, some of them only were classified to class level (Supplementary Table 3). Comparisons with the GTDB showed that one genome in this database “GCA_900319375.1 Rumen Bacteroidales” shared 95% similarity at nucleotide level to one of our bins according to MASH-ANI distances, as shown in Supplementary Figure 1.

**FIGURE 1:**
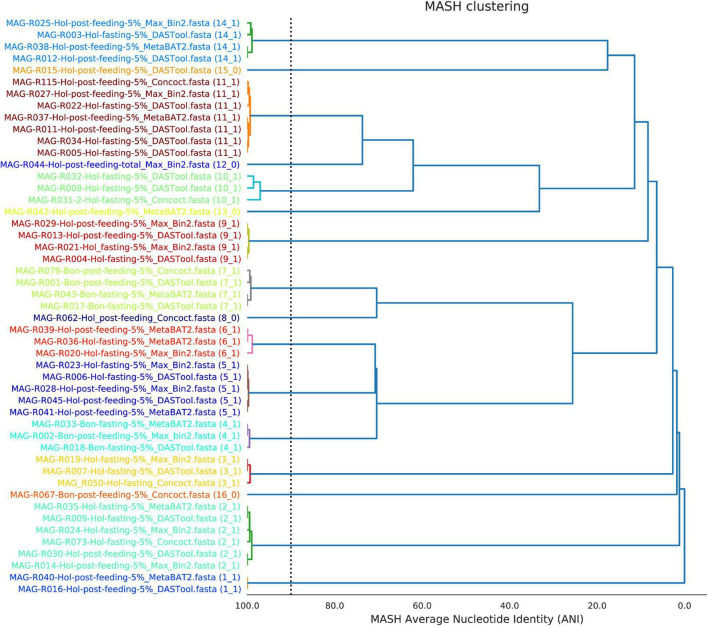
Clustering of the high-quality metagenome-assembled genomes (MAGs) found in the 5% fraction of the total sample of ruminal fluid of the Bon and Holstein animals according to MASH-ANI distances.

The program DREP (Olm et al., 2017) was used to group the bins according to nucleotide average identity and MASH distance or MASH-ANI. Eleven clusters of bins were formed, and five singletons were identified. A representative MAG for each cluster was chosen based on the parameters described for each bin in the MiGA tool, the completeness and contamination value, and the RPKM value. Figure 1 shows a tree based on ANI-MASH distance of all the 49 MAGs resulting in the 16 selected clusters.

Due to the low similarity of our MAGs to publicly available genomes, we compared our MAGs with genomes and MAGs generated in recent rumen studies, by means of AAI (described in section “Materials and Methods”), particularly, the compendium of MAGs of the rumen or RUGs compiled by Stewart et al. (2019) and the complete rumen genomes reported in the Hungate project 1000 database (Seshadri et al., 2018). The most similar genomes to our bins in the RUGs database and the Hungate project 1000 are shown in Supplementary Table 4. In all cases, the AAI values to the best hits in the RUGs database were higher compared with the Hungate 1000 project database, indicating that our bins are similar to MAGs reported in other metagenomic rumen studies than in the cultivated rumen bacteria. An interesting finding is that the genome size of the more similar RUGs is small and similar to the ones obtained for our MAGs, varying from 898,703 to 3,185,451 bp. The completeness and contamination values for the more similar RUGs and their taxonomic classification according to Stewart et al. (2019) are described in Supplementary Table 4, as those genomes were used for further analysis in this study.

### Metagenome-Assembled Genomes Phylogenetic Relatedness

**FIGURE 2:**
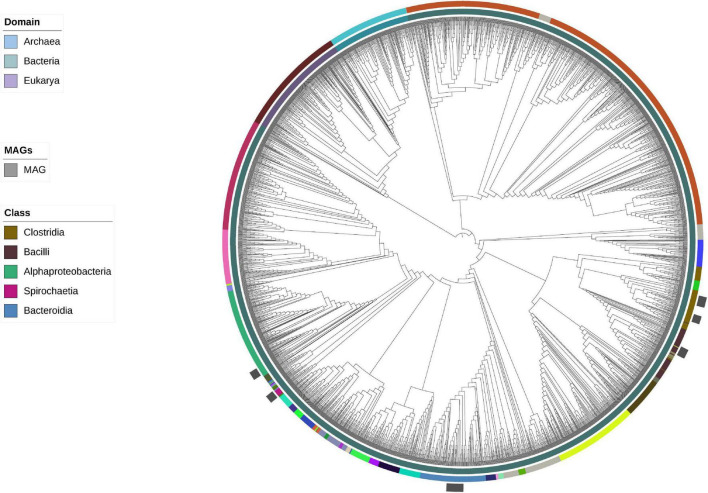
Bootstrapped maximum-likelihood tree based on concatenated ribosomal marker genes including representative MAGs found in this study placed onto the phylogenetic tree proposed by Hug et al. (2016). Clades are colored based on both domain and order, but only orders that are relevant to the MAGs are presented in this study, as shown by gray boxes.

Given that the ANI and AAI metrics suggested that some of the 16 obtained MAGs could correspond to potentially new taxa, a phylogenetic tree was constructed to assess their phylogenetic relatedness. The tree was constructed using concatenated single-copy genes from the MAGs (Figure 2). In addition, a taxonomic assignment was done using phylogenetic placement and RED values using the query genomes in the GTDB reference tree, as implemented in GTDB-Tk (Supplementary Table 5). GTDB-Tk taxonomic assignments placed the MAGs within the following phyla: Firmicutes_A (*n* = 7), Bacteroidota (*n* = 4), Firmicutes (*n* = 2), Proteobacteria (*n* = 2), and Spirochaetes (*n* = 1). All members of Firmicutes_A belonged to the class Clostridia, which included the recently proposed orders 4C28d-15 (*n* = 6) and Christensenellales (*n* = 1). All members of Firmicutes belonged to the class Bacilli; this included the orders RF39 (*n* = 1) and ML615J-28 (*n* = 1). The four Bacteroidota genomes were assigned to the class Bacteroidia and order Bacteroidales, with only one of them assigned to a traditionally described family and genus, that is the Bacteroidaceae and *Prevotella*, respectively. The two Proteobacteria were both assigned to the Class alpha-proteobacteria, and within it to recently proposed orders, Rs-D84 and RF32. The Spirochaetota genome was assigned to the family Sphaerochaetaceae, and the recently proposed genus UBA9732. Only one MAG was assigned to species level (from order Bacteroidales, assigned as RC9 sp900319375), four were not identified to genus (all these Clostridia), and all of them were classified to family and higher taxonomic ranks. It is important to highlight that 10 of these genomes were assigned to recently proposed orders, and 14 to recently described families, particularly within the classes Clostridia, Bacilli, and Alphaproteobacteria, all with names assigned as codes and likely with no cultured representative. No MAGs were identified as Archaea.

### Functional Profile of the Metagenome-Assembled Genomes

In order to analyze the functional potential encoded in our MAGs in the context of other ruminal microorganisms, we compared their predicted functional repertoires with those predicted from the complete genomes of four well-characterized and commonly abundant rumen cultivated bacteria: *Prevotella ruminicola* (GCF_000025925.1), *Prevotella bryantii* (GCF_000179055.1), *Ruminoccocus albus* (GCF_000178155.2), and *Butyrivibrio fibrisolvens* (GCF_000209815.1) (Nathani et al., 2015), as well as the most similar RUGs as evidenced from AAI.

**FIGURE 3:**
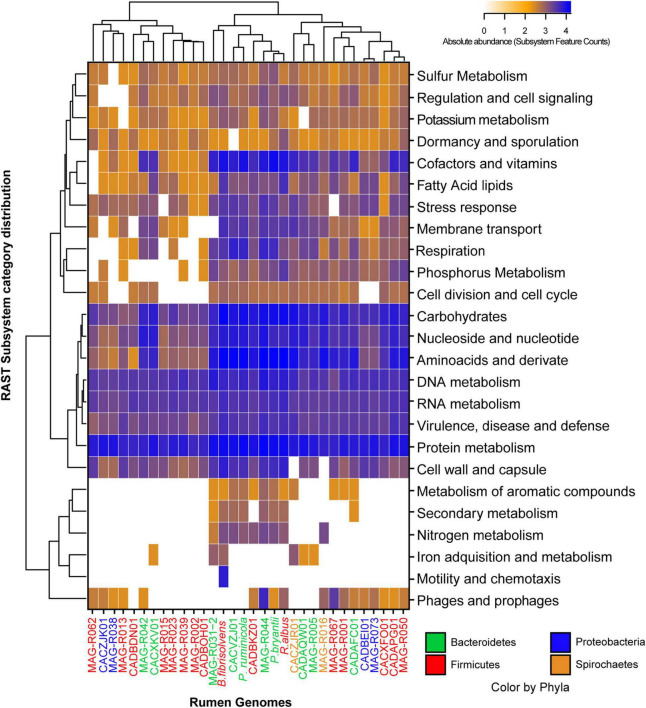
Absolute abundance of the subsystem feature counts found in the functional categories established by RAST (rapid annotation using subsystem technology) for each of the representative MAGs.

In general, the subsystem category distribution with RAST showed that only between 10 and 19% of the proteins of each MAG could be annotated by this tool, which implies that the other proteins are distant homologs from known proteins or new proteins. We further studied all the general functional characterization performed by RAST for all MAGs (Figure 3), the number of genes in each functional category was used to perform a clusterization analysis and build the heatmaps. Three large clusters can be observed based on gene abundance. MAG-R015, MAG-R023, MAG-R039, MAG-R002, MAG-R013, that are members of the phyla Firmicutes_A (Clostridia), MAG-R062 a member of Firmicutes (Bacilli) and MAG-R038 that belongs to Alphaproteobacteria, form one cluster characterized by a smaller number of subsystem feature counts in most of the functional categories and the lack of genes in several categories, including protein degradation. These MAGs seem to have a basic and reduced functional repertoire. Conversely, Bacteroidota genomes, such as MAG-R044 and MAG-R032, form a clade with *P. ruminicola*, *P. bryantii*, *R. albus*, and *B. fibrisolvens.* In addition, these MAGs contain subsystem feature counts in most functional categories, having a greater number of these in the category of carbohydrates, amino acids, and derivatives and in cofactors, and vitamins indicating they have a larger potential metabolic repertoire, at least of characterized genes, than the rest of the MAGs reported in this study. Finally, an intermediate cluster formed with MAG-R005, MAG-R016, MAG-R067, MAG-R001, MAG-R073, and MAG-R050 had a high abundance of genes, related to central metabolism and co-factor and vitamins but absent from certain categories such as the first cluster.

**FIGURE 4:**
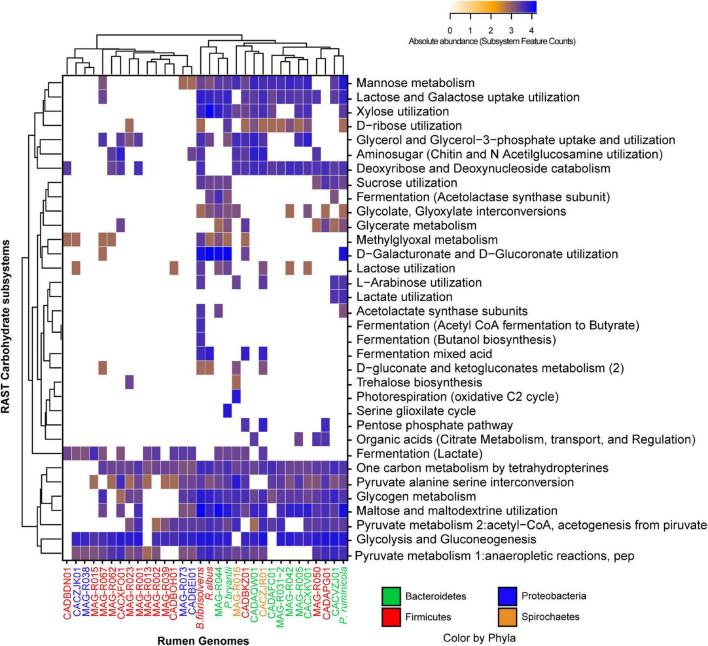
Absolute abundance of the subsystem feature counts found in the carbohydrate category established by RAST (rapid annotation using subsystem technology) for each of the representative MAGs.

After a general functional characterization was performed, we focused on three functional categories: carbohydrate metabolism, vitamins and cofactors, and protein degradation. The degradation of structural carbohydrate from the plant cell wall is an essential process of ruminal microorganisms for obtaining energy. Figure 4 depicts the carbohydrate category present in the MAGs assembled in this study, the Hungate genomes and the RUGs. We observed that some of our MAG and RUG genomes showed very limited capacity for the degradation of complex carbohydrates and oligosaccharides, including MAG-R015, MAG-R067, MAG-R023, MAG-R039 and MAG-R002 belonging to Firmicutes_A (Clostridia), MAG-R062 belonging to Firmicutes (Bacilli), MAG-R073, MAG-R038 belonging to Alphaproteobacteria; however, most of these MAGs have the genes that will allow them to ferment lactate. On the other hand, MAG-R044 has the largest metabolic repertoire for the potential degradation of complex polysaccharides such as cellulose, hemicellulose, and pectin and its derived oligosaccharides, and it is important to highlight that this MAG was the only one that was enriched in the total and not the 5% fraction of the gradient. Other Bacteroidota, such as MAG-R031-2, MAG-R042, and MAG-R005, showed fewer carbohydrate degradation genes than MAG-R044, but they can also degrade oligosaccharides, and only MAG-R005 can degrade xylose. Spirochaete MAG-R016 has the potential to degrade arabinose, a monosaccharide derived from hemicellulose degradation. Most of the MAGs present subsystem feature counts for glycolysis and gluconeogenesis.

**FIGURE 5:**
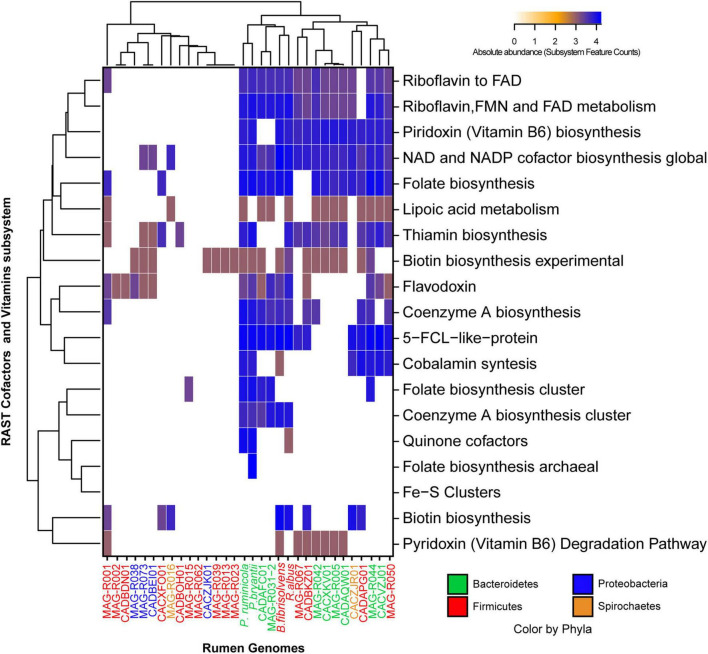
Absolute abundance of the subsystem feature counts found in the vitamins and co-factors category established by RAST (rapid annotation using subsystem technology) for each of the representative MAGs.

**FIGURE 6:**
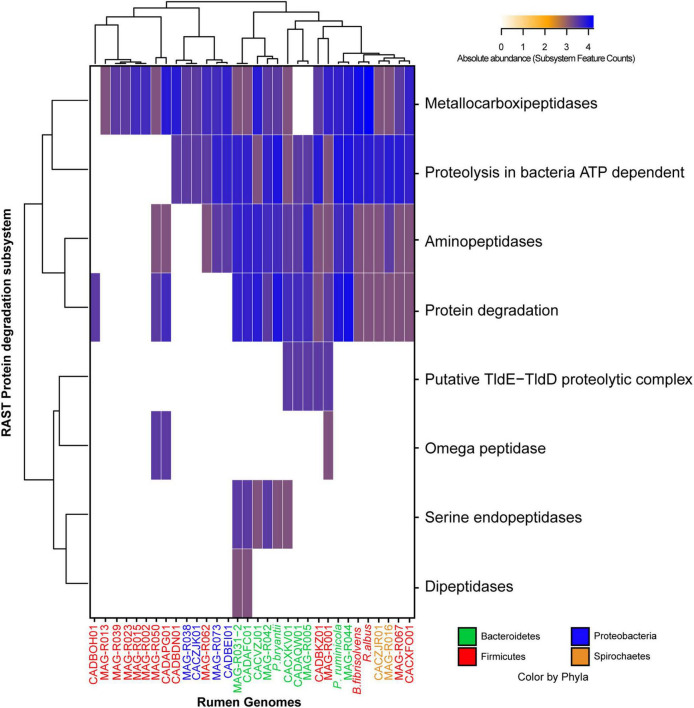
Absolute abundance of the subsystem feature counts found in the protein degradation category established by RAST (rapid annotation using subsystem technology) for each of the representative MAGs.

MAGs were also clustered according to the presence and abundance of genes associated with the vitamin and co-factor categories: the cluster formed by Alphaproteobacteria (MAG-R038 and MAG-R073), Spirochaetes (MAG-R016), most of the MAGs belonging to Firmicutes_A; Clostridia (MAG-R002, MAG-R015, MAG-R039, MAG-R013, and MAG-R0 23) and Firmicutes; Bacilli (MAG-R062 and MAG-R001) had a few genes related with vitamins and co-factors (Figure 5). In contrast, the clusters of bins represented by all the Bacteroidota and MAG-R050 and MAG-R067, which belong to Firmicutes phylum, contain predicted genes for the synthesis of most of the vitamin B cluster. We also evaluated the proteolytic activity of the assembled MAGs using the annotation of the subcategory of protein degradation, which is part of the functional category of protein metabolism in RAST. Protein degradation is essential for ammonia production by the rumen microbial community (Lu et al., 2019). All the MAGs assembled in this study have metallocarboxypeptidases (Figure 6). These types of enzymes free an amino acid at the carboxy terminal of a polypeptide and depend on a metal ion to work properly (Fernandes et al., 2021). These proteinase genes were the only ones in the MAGs that belong to Firmicutes_A Clostridia, such as the MAG-R013, MAG-R039, MAG-R023, MAG-R015, and MAG-R050. Other types of enzymes, such as the aminopeptidases, which release the amino-terminal amino acid from a polypeptide (Gonzales and Robert-Baudouy, 1996), were also detected in all the Bacteroidota, Spirochaetaceae, Firmicutes, Bacilli, and Alphaproteobacteria MAGs, while enzymes, such as serine-aminopeptidases and dipeptidase were detected only in the Bacteroidota phylum. The ATP-dependent proteolysis in bacteria is present in most of the MAGs assembled in this study except for most of the Firmicutes_A clostridia. This function is related to the recognition, unfolding, and breaking of bonds of a protein that lead to the formation of large peptides (Baker and Sauer, 2006).

### The Degradation of Complex Polysaccharides: Carbohydrate-Active Enzymes

**FIGURE 7:**
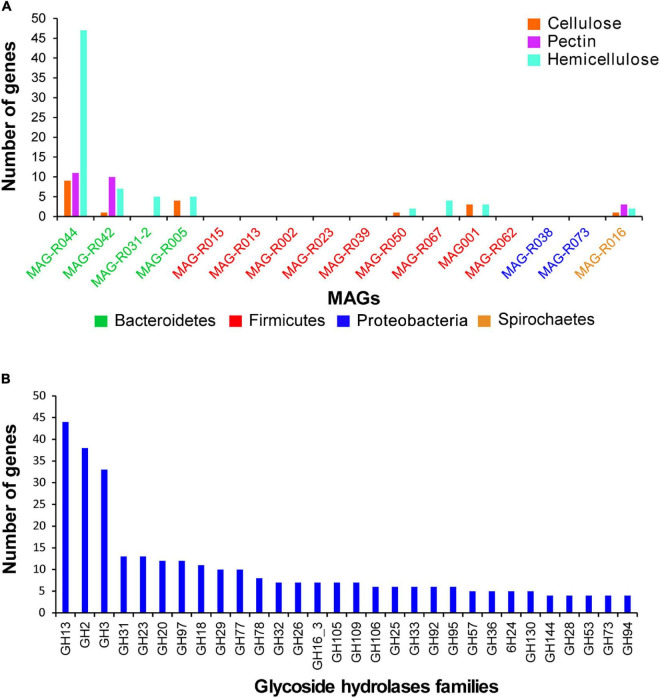
Glycoside hydrolases found in the representative MAGs using dbCAN2 carbohydrate-active enzyme (CAZy) domain HMM database. **(A)** Gene counts per each MAG of enzymes degrading substrates such as cellulose, hemicellulose, and pectin, according to Seshadri et al. (2018). **(B)** Gene counts for other glycoside hydrolases.

The dbCAN2 metaserver (Zhang et al., 2018) was used to search for carbohydrate-active enzymes in the MAGs. The presence of the enzymes that degrade cellulose, hemicellulose, and pectin in the rumen was established by those families of carbohydrate active enzymes previously reported (Seshadri et al., 2018) as follows: cellulose degradation: GH5, GH9, GH44, GH45, and GH48; xylan degradation: GH8, GH10, GH11, GH43, GH51, GH67, GH115, GH120, GH127, CE1, and CE2; pectin degradation: GH28, PL1, PL9, PL10, PL11, CE8, and CE12. Figure 7A shows the number of genes that degrade cellulose, hemicellulose, and pectin in the MAGs reconstructed in this study. In this sense, all the MAGs that belong to Bacteroidetes, Spirochaetes, MAG-R050, MAG-R067, and MAG-R001 belonging to Firmicutes have genes for the degradation of these complex structural carbohydrates. Only MAG-R044, MAG-R042, and MAG-R016 have genes for the degradation of cellulose, pectin, and hemicellulose. MAG-R044 has the largest number of genes that degrade these complex structural carbohydrates. On the contrary, no related genes to the degradation of cellulose, hemicellulose, or pectin were found for eight MAGs belonging to Clostridia (Firmicutes_A) and Alphaproteobacteria (Supplementary Table 6A).

Figure 7B shows other glycoside hydrolases families found in the reconstructed MAGs. The most abundant families of glycoside hydrolases found for all were family GH13, which degrades starch. Other abundant families were GH2, GH3, GH31, GH20, GH97, and GH18, which degrade substrates such as beta-galactosidases, cellobiose and xylan, maltose, or heptomaltose, peptidoglycans, N-acetyl galactosamine, and chitin (Supplementary Table 6B). All these compounds are present in the ruminal environment; some of them are oligosaccharides derived from the degradation of cellulose, pectin, and hemicellulose, and others, such as peptidoglycans and chitin, are found in the bacterial cell wall and in ruminal fungi. The Bacteroidota phyla showed most of the families of hydrolases found in this study, while most of the Clostridia MAGs have genes of the family GH23, which degrades peptidoglycan and chitin dextrins.

Other esterases also were described in all the MAGs. Supplementary Table 6C shows the number of genes for each esterase family in each of the bins. In general, CE4 was the most abundant family. This family is enriched mostly in the Firmicutes_A Clostridia MAGs. Described substrates for this family are xylan, chitin, chitooligosaccharide, and peptidoglycans. The transferase families found in the reconstructed MAGs are shown in the Supplementary Table 6D. The most abundant transferase family is the GT2 followed by other transferase families, such as GT4, GT51, and GT28. All these families are present in most of the MAGs assembled in this study. In addition, a higher number of transferase families’ genes were found in Clostridia MAGs, while in Bacteroidota MAGs, a higher number of hydrolase families’ genes were counted (Supplementary Figure 2). Other enzymes, such as CBMs, polysaccharide lyases, and auxiliary activity enzymes were found in the MAGS in very low abundance. These enzymes are reported in Supplementary Tables 6E–G.

## Discussion

The taxonomic and functional characterization of the microorganisms that are part of the ruminal ecosystem is an essential factor to understand the complex microbial interactions that occur when plant fiber is fermented. This characterization is also fundamental to elucidate how to improve cattle’s health, productivity, and to search for methods to decrease methane emissions (Stewart et al., 2019). However, the best characterized genomes at the taxonomic and functional level are genomes belonging to the most abundant microorganisms in the rumen, leaving aside other microorganisms (Eisenstein, 2018). In this study, we reconstructed the genomes of 15 species enriched by fractionation and one genome from the total fraction, from two cattle breeds that grazed tropical grasses. These 15 genomes were enriched by fractionating the rumen microbial diversity by size and density, using a sucrose density gradient, a methodological approach developed recently by Hernández et al. (2021).

The fractionation of ruminal samples by size and density allowed the enrichment of bacteria present in the rumen for further functional and taxonomic identification. It was possible to see a consistency in terms of the MAG presence in each animal, with its presence identified both in the 5% fraction and the total samples. However, in 15 out of the 16 cases, the MAGs were more abundant in the fractions than the total sample, showing that the enrichment process reduces the complexity of the sample, allowing a more efficient assembly. Further proof of this comes from the higher percentage of read recruitment from the 5% fractions than the total samples to the MAGs assembled. The low percentage of reads mapping to the enriched MAGs in the unfractionated samples suggests that those assembled genomes would have been very difficult to assemble unless a similar enrichment was done, or a significantly higher depth of sequencing was achieved. The high diversity and abundance variability of ruminal communities imply that there are large fractions of poorly characterized microorganisms, but some studies performed in the human gut are elucidating the importance of these microorganisms (Almeida et al., 2019). New functional genes relating the carbohydrate metabolism with antimicrobial, and biotechnology applications were found in the rare bacterial genomes in different human populations. These genes were not present in the genomes of the most abundant bacteria in the human gut or in those microorganisms that have a culture-isolated representative (Almeida et al., 2019).

An interesting property of most MAGs recovered from the 5% fraction enrichment is their small genome size. Other rumen researchers have reported small genome sizes MAGs (Seshadri et al., 2018). According to Duda et al. (2012), a small bacterial genome size varies between 0.58 and 3.2 Mbp. This small genome size agrees with the bacterial size in the 5% fractions ranging from 0.3 to 0.7 μm^2^ (Hernández et al., 2021). In general, the presence of small-sized bacteria in an environment is due to nutrient shortage, and when the availability of nutrients increases, the size of the bacteria also increases (Ghuneim et al., 2018). Several factors can trigger small-sized bacteria in an ecosystem; for example, protozoan grazing, as occurs in the rumen, can induce large small-sized bacterial populations due to protozoa usually feeding on bacteria with a size between 0.8 and 4 μm; thus, bacteria with a smaller size can proliferate easily. Another factor is the lysis of bacterial cells produced by bacteriophages, which release nutrients or public goods to the rumen environment, which are available for other members of the microbial community, such as small bacteria with reduced metabolic pathways (Ghuneim et al., 2018). The ease of availability of nutrients can lead to a loss of genes and a reduction in the genome size, an evolutionary process that could have associated to small-sized bacteria (Morris et al., 2012) (Another reason for small, detected genome size is a process of genome reduction which can happen in certain symbionts and obligate parasites). The genome reduction can happen in certain symbionts and obligate parasites (Ghuneim et al., 2018).

We achieved the assembly and characterization of MAGs belonging to different phyla, such as Bacteroidota, Firmicutes_A, Firmicutes, Proteobacteria, and Spirochaetota. Fifteen MAGs were novel at the species level, and four at the genus level. Furthermore, even though all MAGs could be assigned at the order and family levels, very few were assigned to a well-described order (7) or family (2). Together, these results highlight that fractionation is an efficient method for enriching bacteria from poorly characterized taxa, at enough abundance to allow genome reconstruction and, thus, better phylogenetic placement. As was observed in our results, only a few MAGs were classified to traditional, well-described bacterial orders and families, and most of the currently proposed taxa are derived precisely from MAGs and other efforts for genome enrichment and assembly, such as the one described here, thus, highlighting the importance of initiative such as the one reported.

The MAGs that belong to the Bacteroidota phylum possess the highest number of glycoside hydrolases that degrade cellulose, xylan, pectin, starch, and oligosaccharides, suggesting that one of the roles of the Bacteroidota MAGs is the degradation of complex and simple polysaccharides derived from plant material in the rumen. Together, with the fact that Firmicutes were usually the most commonly studied ruminal microorganisms for polysaccharide degradation, our results highlight the potential of the enrichment method as a previous step toward culture-based efforts directed toward isolation of novel Bacteroidota. These results agree with those reported in previous investigations about Bacteroidota in the rumen. In these studies, Bacteroidota is associated with saccharolytic activities, cellulose degradation through polysaccharides utilization loci (PUL’s) (Naas et al., 2014) and the degradation of xylan (Dodd et al., 2010). Bacteroidota MAGs were also characterized by the synthesis of different vitamins, such as B7, B1, B9, B2, and B12. These vitamins participate in different ruminal functions, such as the metabolism of amino acids, lipids, and carbohydrates, amino acid catabolism, one carbon metabolism, methionine synthesis, and co-factors in essential proteins that support cellular function in the microbial community of the rumen and in the host animal (Seck et al., 2017). These vitamins also have a role in DNA synthesis, such as the B12 vitamin, which participates in the synthesis of DNA and in essential proteins (Kaur et al., 2019). High levels of B12 vitamins are related to the abundance of the *Prevotella* genus in the rumen (Franco-Lopez et al., 2020). The MAG-R044 that was classified as the genus *Prevotella* supports this finding due to the presence of Cobalamin biosynthesis genes. Interestingly, the MAG-R050 belonging to Firmicutes also has this metabolic potential. Although most MAGs belonging to Firmicutes_A have few genes for vitamin synthesis, exceptions are biotin biosynthesis and flavodoxin. Biotin is an important vitamin for cellulose degradation, the production of volatile fatty acids in the rumen (Milligan et al., 1967), and for animal metabolism (Chen et al., 2011). Flavodoxin is an electron-carrying protein in bacteria (Yoch and Valentine, 1972) linked to the pyruvate pathway necessary for the volatile fatty acid production by microorganisms in the rumen and NADH oxidation (Hart et al., 2018).

*Ruminococcus* and *Butyrivibrio* are representative genera of Firmicutes_A in the rumen; their main function is the digestion of cellulose, xylan, and pectins (Klieve et al., 2005; Palevich et al., 2019). According to the CAZy annotation, only MAG-R050, MAG-R067, and MAG-R001 possess glycoside hydrolases for cellulose and hemicellulose degradation. Most of the Firmicutes_A, Firmicutes Bacilli, and an Alphaproteobacteria MAGs reconstructed in this study are characterized by the absence or very few genes for glycoside hydrolases. These results are supported by the RAST carbohydrate function characterization where one or few of them have genes for lactose utilization, mannose metabolism, chitin, and N-acetyl-glucosamine utilization. Comparable results were observed in other MAGs reconstructed in other rumen studies such as CADBN01, CACXFO01, and CADBOH01 (Figure 4). However, most of the Firmicutes MAGs have genes associated with the production of lactate, which is an intermediate compound in the production of volatile fatty acid necessary for cattle metabolism and is converted to propionic acid and propionate by other rumen microorganisms like *Megasphaera elsdenii* (Counotte et al., 1983). The genes to produce lactate were also observed in the Alphaproteobacteria MAGs.

Most of the Firmicutes MAGs also were characterized by the absence of glycoside hydrolases, vitamins, and co-factor genes, as well as a few protein-degradation genes. This general lack of pathways could be due to the incomplete reconstruction of these MAGs or lack of known homologs that allow for the annotation of such functional genes in their genomes. The lowest completeness values among Firmicutes MAGs were 69.09% (MAG-R015) and 71.50% (MAG-67). The rest of the MAGs have a completeness value greater than 80%; in fact MAG-R001, MAG-R012, MAG-R039, and MAG-R050 have completeness values greater than 90–96.61%. In addition, the RUGs that have a protein composition similar to our MAGs also showed a reduction in the metabolic pathways, although they have high completeness values between 84.36 and 95.75%. Assuming that the functional reduction is not a technical artifact, another explanation to understand the reduction in metabolic pathways in Firmicutes is genome size reduction where these bacteria depend on other rumen microorganisms to supply metabolic requirements (Morris et al., 2012). These results are supported by previous research where some bacteria from Firmicutes and Proteobacteria phylum have lost some families of glycoside hydrolases, the glycogen pathways, and other enzymes such as enolases that participate in glycolysis. These bacteria feed from the amino acids and volatile fatty acids generated by primary fermenters in the rumen (Seshadri et al., 2018).

On the other hand, our findings suggest that some Firmicutes could have a specialized niche. Most of the Firmicutes_A MAGs showed an enrichment of the family CE4; this family of esterases deacetylate xylan, chitin, chitooligosaccharides, and peptidoglycans. In addition, genes of the GH23 and GH18 families were also found in this taxonomic group. The function of the family GH23 is a peptidoglycan and chitin lysozyme, while family GH18 has, among other functions, the activity of chitinase. Chitin is a component of the fungal cell wall (Lenardon et al., 2010), and anaerobic fungi are part of the rumen microbial community. According to these findings, chitin can be potentially used by Firmicutes MAGs as a source of energy, and so, they contribute to degradation and recycling of chitin in the rumen. Chitinolytic bacteria have been reported previously in the rumen. Kopeèný et al. (1996) grew chitinolytic bacteria isolated from cow and sheep feces in a rumen fluid medium with colloidal chitin. The taxonomic classification of these bacteria was *Clostridium* sp. (based on physiological and biochemical tests); these bacteria were reported to produce acetate, butyrate, and lactate from chitin fermentation. On the other hand, the presence of lysozyme could indicate that these enzymes are involved in the cleavage of the bacterial cell wall (peptidoglycan), for cell division and turnover, or even peptidoglycan can be used as an energy source (Park and Uehara, 2008), and not for chitin degradation (Blackburn and Clarke, 2001). Further research will be needed to explain in detail these relationships between rumen organisms.

The large number of transferases found in Firmicutes MAGs also suggests that they are bacteria that can be specialists in producing polysaccharides. The GT2 transferase family genes are present not only in Firmicutes but in most of the reconstructed MAGs in this study. An important function of this gene family is the cellulose synthesis, although the presence of this gene family does not guarantee the production of cellulose by the microorganism, and close homologs to other cellulose synthesis genes was not significantly detected, it has been reported that the bacterial cellulose is produced by environmental bacteria, and the most common role is the attachment of a bacterial cell to other bacterial cells, to the host or to a substrate (Ross et al., 1991). In this sense, cellulose, although a rare component of biofilm, could be important for its formation (Römling and Galperin, 2015). In the rumen, the biofilm formation is an essential process to degrade the vegetal fiber where primary colonizer bacteria, which degrade cellulose, pectin, and hemicellulose, form a biofilm with secondary colonizer bacteria increasing the degradation rate of these complex polysaccharides (McAllister et al., 1994). In consequence, the presence of cellulose synthesis-related genes is an interesting finding, and further studies would be required in order to prove that the complete gene repertoire needed for cellulose synthesis is present. This is an important point since bacterial cellulose has multiple industrial applications (Wang et al., 2019).

In conclusion, the enrichment of specific bacteria from the rumen, using a sucrose-based fractionation is a powerful methodology that allows us to characterize, both phylogenetically and functionally, small-sized bacterial genomes. These bacteria with small genomes could not be assembled using only the standard/total sample of ruminal fluid. We were able to reconstruct the genomes of 16 MAGs from Colombian creole breeds that live in tropical environments. Phylogenetic placement in the tree of life reveals that 15 MAGs are new species, which belong to the Bacteroidota, Firmicutes, Firmicutes_A, Alphaproteobacteria, and Spirochaetota phyla. These results add to the knowledge of the enormous bacterial diversity that has not yet been discovered in the ruminal ecosystem. Despite its small genome size, Bacteroidota and Spirochaetota species showed a wide potential functional diversity adapted to the hydrolysis of complex polysaccharides in the plant cell wall and toward the production of B-complex vitamins and protein degradation in the rumen. Conversely, the MAGs belonging to Firmicutes, Firmicutes_A, and Alphaproteobacteria showed a reduction in several metabolic pathways either due to an incomplete reconstruction of these MAGs or that they went through a process of genome size reduction generated by protozoa predation and availability of nutrients that leads them to depend on other microorganisms that inhabit the rumen to supply their metabolic requirements from primary fermenters. Only between 10 and 19% of the proteins of each MAG could be annotated by RAST in these genomes; therefore, there is still an enormous functional potential that needs to be further explored to fully characterize the functional peculiarity of these genomes in the rumen.

Tools such as fractionation or selection of small-sized microorganisms in an environment, such as the rumen, have the potential to unveil genomic characteristics and functional roles overseen when studying a complete sample of such an environment. In our case, it revealed genomic factors from novel representatives of phyla, such as Firmicutes and Bacteroidetes, that are not necessarily the most common features from cultured representatives of those phyla. Furthermore, it suggests that at all taxonomic levels, the functional partitioning of an environment, such as the rumen, is much more complex that could be explored from the abundant members. Our study showed that we were able to enrich and characterize genomic and functional features of organisms from poorly characterized or novel taxa. Finally, this type of fractionation and selection can constitute the first step in isolating and recovering such microorganisms with their corresponding biotechnological and agricultural potential.

## Data Availability Statement

The datasets presented in this study can be found in online repositories. The names of the repository/repositories and accession number(s) can be found below: https://www.ebi.ac.uk/ena, European Nucleotide Archive (ENA), project accession PRJEB47520.

## Ethics Statement

Ethical review and approval was not required for the animal study because although the original samples were obtained from bovine rumen, the current research is based on samples from a sucrose gradient fractionation derived from the original samples. Description and ethical consent for the original samples is provided in the original publication also in Frontiers in Microbiology (doi: 10.3389/fmicb.2021.664754).

## Author Contributions

AC-Q, AR, HJ, and RH contributed to the design and supervision of this study. AC-Q and HJ provided material and resources for sampling and the processing of samples in the lab. RH performed the sampling and the sample processing and drafted the first draft of the manuscript. AR provided the computational resources. RH and MC mainly performed bioinformatic analyses. MC wrote the phylogenetic methods and results. All authors contributed to manuscript revision and approved it for publication.

## Conflict of Interest

The authors declare that the research was conducted in the absence of any commercial or financial relationships that could be construed as a potential conflict of interest.

## Publisher’s Note

All claims expressed in this article are solely those of the authors and do not necessarily represent those of their affiliated organizations, or those of the publisher, the editors and the reviewers. Any product that may be evaluated in this article, or claim that may be made by its manufacturer, is not guaranteed or endorsed by the publisher.

## Footnotes

1
https://merenlab.org/2016/06/22/anvio-tutorial-v2/

2
https://gtdb.ecogenomic.org/

3
https://github.com/dparks1134/CompareM
