# Supplementary material for: Functional and Phylogenetic Characterization of Bacteria in Bovine Rumen Using Fractionation of Ruminal Fluid
Source: Front Microbiol. 2022 Mar 25;13:813002. doi: 10.3389/fmicb.2022.813002 (PMC8992543; doi:10.3389/fmicb.2022.813002)
Supplement: Supplementary file 1 [file Image_1.pdf]

Supplementary Material

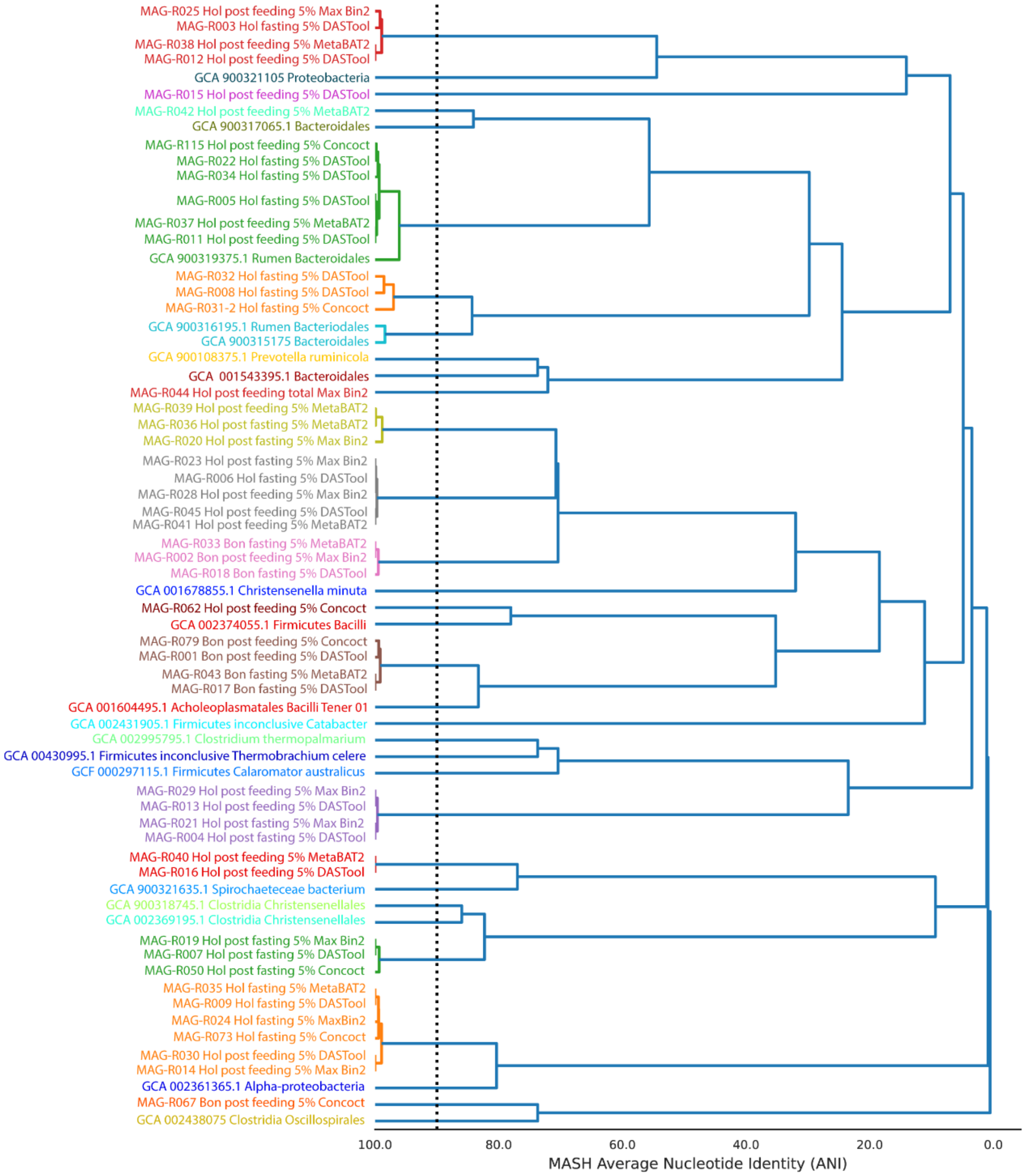

Supplementary figure 1. Clustering of the high-quality MAGs assembled in the 5% fraction and the total sample of ruminal fluid of the Bon and Holstein animals, and the most similar genomes found in the Genome taxonomy Database according to MASH-ANI distances.

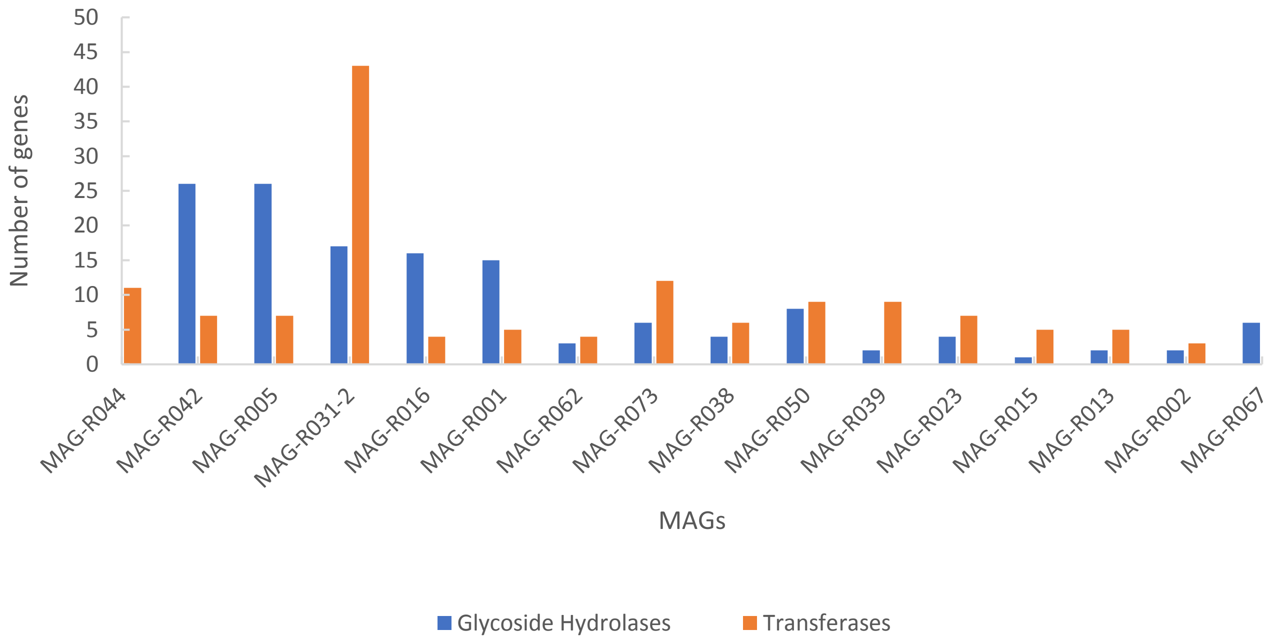

Supplementary figure 2. Number of Glycoside hydrolases and transferases found in the Representative MAGs.
